# Internalizing and externalizing symptoms in children during the COVID-19 pandemic: a systematic mixed studies review

**DOI:** 10.3389/fpsyg.2023.1182309

**Published:** 2023-06-15

**Authors:** Annalisa Levante, Chiara Martis, Federica Bianco, Ilaria Castelli, Serena Petrocchi, Flavia Lecciso

**Affiliations:** ^1^Department of Human and Social Sciences, University of Salento, Lecce, Italy; ^2^Lab of Applied Psychology, Department of Human and Social Sciences, University of Salento, Lecce, Italy; ^3^Department of Human and Social Science, University of Bergamo, Bergamo, Italy; ^4^Faculty of Biomedical Sciences, Università della Svizzera Italiana, Lugano, Switzerland

**Keywords:** COVID-19, child, internalizing symptoms, externalizing symptoms, systematic mixed studies review, MMAT

## Abstract

**Introduction:**

Given the vulnerability of children during the COVID-19 pandemic, paying close attention to their wellbeing at the time is warranted. The present protocol-based systematic mixed-studies review examines papers published during 2020–2022, focusing on the impact of the COVID-19 pandemic on children's internalizing/externalizing symptoms and the determinants thereof.

**Method:**

PROSPERO: CRD42022385284. Five databases were searched and the PRISMA diagram was applied. The inclusion criteria were: papers published in English in peer-reviewed journals; papers published between January 2020 and October 2022 involving children aged 5–13 years; qualitative, quantitative, and mixed studies. The standardized Mixed Method Appraisal Tool protocol was used to appraise the quality of the studies.

**Results:**

Thirty-four studies involving 40,976 participants in total were analyzed. Their principal characteristics were tabulated. The results showed that children's internalizing/externalizing symptoms increased during the pandemic, largely as a result of disengagement from play activities and excessive use of the internet. Girls showed more internalizing symptoms and boys more externalizing symptoms. Distress was the strongest parental factor mediating children's internalizing/externalizing symptoms. The quality of the studies was appraised as low (*n* = 12), medium (*n* = 12), and high (*n* = 10).

**Conclusion:**

Gender-based interventions should be designed for children and parents. The studies reviewed were cross-sectional, so long-term patterns and outcomes could not be predicted. Future researchers might consider a longitudinal approach to determine the long-term effects of the pandemic on children's internalizing and externalizing symptoms.

**Systematic review registration:**

https://www.crd.york.ac.uk/prospero/display_record.php?ID=CRD42022385284, identifier: CRD42022385284.

## 1. Introduction

In most countries, the main restrictive measures applied to control the spread of COVID-19 between 2020 and 2022 included total lockdowns (or “sheltering in place” in North America), the closure of educational institutions and workplaces, social isolation, and prohibitions on gatherings. These restrictions significantly affected mental health within the general population (Hossain et al., 2020)—for instance, high levels of stress amongst health care professionals (Batra et al., 2020; Muller et al., 2020; Newby et al., 2020; Franklin and Gkiouleka, 2021) and low levels of wellbeing and even burnout amongst educators (partly as a response to the shift from one-to-one to remote teaching; Chan et al., 2021; Ozamiz-Etxebarria et al., 2021; Levante et al., 2023). Meanwhile, many parents found it difficult to balance work and family life (Graham et al., 2021). Because schools were closed, they were obliged to serve a range of roles (e.g., those of educator, caregiver, and playmate) for their typically developing children (Spinelli et al., 2020; Sun et al., 2022) or their atypically developing children (Levante et al., 2021; Calderwood et al., 2022).

Confinement and uncertainty (Petrocchi et al., 2022) had detrimental effects on several aspects of children's psychological functioning, such as high levels of anxiety (Orgilés Amorós et al., 2021; Aras Kemer, 2022), depression (Duan et al., 2020; Orgilés Amorós et al., 2021), hyperactivity and peer issues (Ravens-Sieberer et al., 2022), attention problems, aggressive behaviors (Khoury et al., 2021), and nervousness and irritability (Mariani Wigley et al., 2021). Some children developed sleep problems (Fidanci et al., 2021), insomnia (Bacaro et al., 2021), and eating disorders (Capra et al., 2023) consequent upon the disruption of their daily routines. Although COVID-19 was acknowledged by the authorities to pose a minute risk to children (Shekerdemian et al., 2020), previous literature has shown that they are more vulnerable to stress and low levels of wellbeing during emergencies and disasters (Danese et al., 2020; Raccanello and Vicentini, 2022).

In light of the above, we decided to carry out a systematic review of empirical studies investigating the impact of the pandemic on children's mental health. We focused on middle childhood, which encompasses the ages 5–13 years. According to Erikson's psychosocial model (Erikson, 1993), the circle of influence on children widens during this period (largely as a result of going to school and social interactions generally). When children have satisfactory social relationships (i.e., they develop a sense of industry), they perform developmental tasks successfully; when they do not, they are at risk of developing emotional and behavioral problems (e.g., a sense of inferiority; Erikson, 1993).

During the pandemic, children were compelled to forsake in-person social interactions for prolonged periods, and a pattern of internalizing and externalizing symptoms emerged (Nivard et al., 2017). Internalizing symptoms are an expression of an individual's internal distress (e.g., trait anxiety and depression; Cosgrove et al., 2011). Externalizing symptoms are expressed outwardly (e.g., aggression, defiance, and behavioral problems; Cosgrove et al., 2011). Pre-pandemic evidence (Bukowski and Adams, 2005; Laursen et al., 2007) revealed that social isolation during middle childhood negatively affected the mental health of adolescents and young adults (e.g., in terms of depression, anxiety, aggression, and anger).

To the best of our knowledge, four systematic reviews of empirical studies carried out during the pandemic have been published, primarily evaluating the mental health of children overall. Ma et al. (2021) measured the impact of the COVID-19 pandemic on children's and adolescents' depressive symptoms, trait anxiety, sleep problems, and post-traumatic stress symptoms. The three others (Aarah-Bapuah et al., 2022; Amorós-Reche et al., 2022; Ng and Ng, 2022) explored participants' emotional and mood problems (Amorós-Reche et al., 2022), depressive symptoms and anxiety (Aarah-Bapuah et al., 2022; Amorós-Reche et al., 2022; Ng and Ng, 2022), withdrawal (Ng and Ng, 2022), and anger and irritability (Ng and Ng, 2022).

Although these reviews provide valuable information, they have certain limitations. Amorós-Reche et al. (2022) and Ng and Ng (2022) examined studies published over 2 years (i.e., 2020–2021), but the other two papers (Ma et al., 2021; Aarah-Bapuah et al., 2022) covered only 6–9 months. Ma et al. (2021) and Amorós-Reche et al. (2022) limited their electronic searches to studies carried out in Spain and China/Turkey, respectively. The most recent review, by Ng and Ng (2022), extracted data from papers published up to February 2022 but did not include mixed studies carried out during the pandemic. Given that the impact of the latter on children's mental health is an exponentially growing field of research, an updated systematic review summarizing the results published thus far on children's internalizing/externalizing symptoms during the period is needed. The present study systematically extracted and reviewed studies that used qualitative, quantitative, and mixed study designs and applied a narrative approach to synthesize the findings in accordance with a standardized protocol. The research questions were formulated according to the PEO format. We extracted papers on typically developing children (**P**opulation) carried out during the pandemic (**E**xposure) that investigated their internalizing and externalizing symptoms (**O**utcomes), then formulated the following research questions:

**RQ1**: What was the impact of the COVID-19 pandemic on children's internalizing/externalizing symptoms?
**RQ2**: What psychological determinants were associated with or contributed to their internalizing/externalizing symptoms?
**RQ3**: Were there any gender differences in terms of children's internalizing/externalizing symptoms?
**RQ4**: Did any parent-related psychological determinants associate with or contribute to children's internalizing/externalizing symptoms?

## 2. Methods and materials

The review protocol was pre-registered on PROSPERO (Protocol No. CRD42022385284).

### 2.1. Search strategy

To extract the studies for review, we applied the updated Preferred Reporting Items for Systematic Review and Meta-Analysis (PRISMA) diagram (Page et al., 2021). An initial electronic search of MEDLINE, PsycINFO, CINAHL, SCOPUS, and Web of Science was carried out in October 2022. In accordance with the PEO format, the keywords and MeSH terms were combined using the Boolean operators AND and OR: child^*^, OR children AND COVID^*^ OR coronavirus OR corona OR COVID-19 OR COVID19 OR COVID OR SARS-CoV-2 OR SARSCoV-2 OR novel coronavirus OR SARS virus OR pandemic OR severe acute respiratory syndrome AND internal^*^ OR external^*^ OR emotion^*^ OR behav^*^.

We confined the studies written in English in the fields of psychology, social science, and health but did not impose restrictions on the countries in which the studies were carried out. The inclusion criteria were: (a) participants recruited from the general population; (b) participants aged ≥5 and ≤ 13 years; (c) papers published in peer-reviewed journals; (d) papers published between January 1, 2020 and the end of October 2022; (e) papers based on COVID-19-related effects; and (f) qualitative, quantitative, and mixed study designs. The exclusion criteria were: (a) participants aged ≤ 4 and ≥14 years; (b) papers that did not report the participants' age; (c) papers from other research fields (e.g., medicine; biology); (d) dissertations, conference abstracts and/or papers, editorials, opinions, commentaries, recommendations, letters, books, and book chapters; (e) other systematic and non-systematic reviews; and (f) validation studies.

### 2.2. Selection of the studies

The PICOS (Bowling and Ebrahim, 2005; Hong et al., 2018) protocol was used to analyze the content of the studies.

**P**articipants: typically developing children aged 5–13 years;
**I**ntervention: studies assessing children's internalizing and externalizing symptoms during the COVID-19 pandemic;
**C**omparison: Gender differences between symptoms;
**O**utcomes: levels of children's internalizing and externalizing symptoms; children's psychological determinants associated with the Intervention variables; parental psychological determinants associated with or contributing to the Intervention variables.
**S**tudy: quantitative; qualitative; mixed.

**Figure 1:**
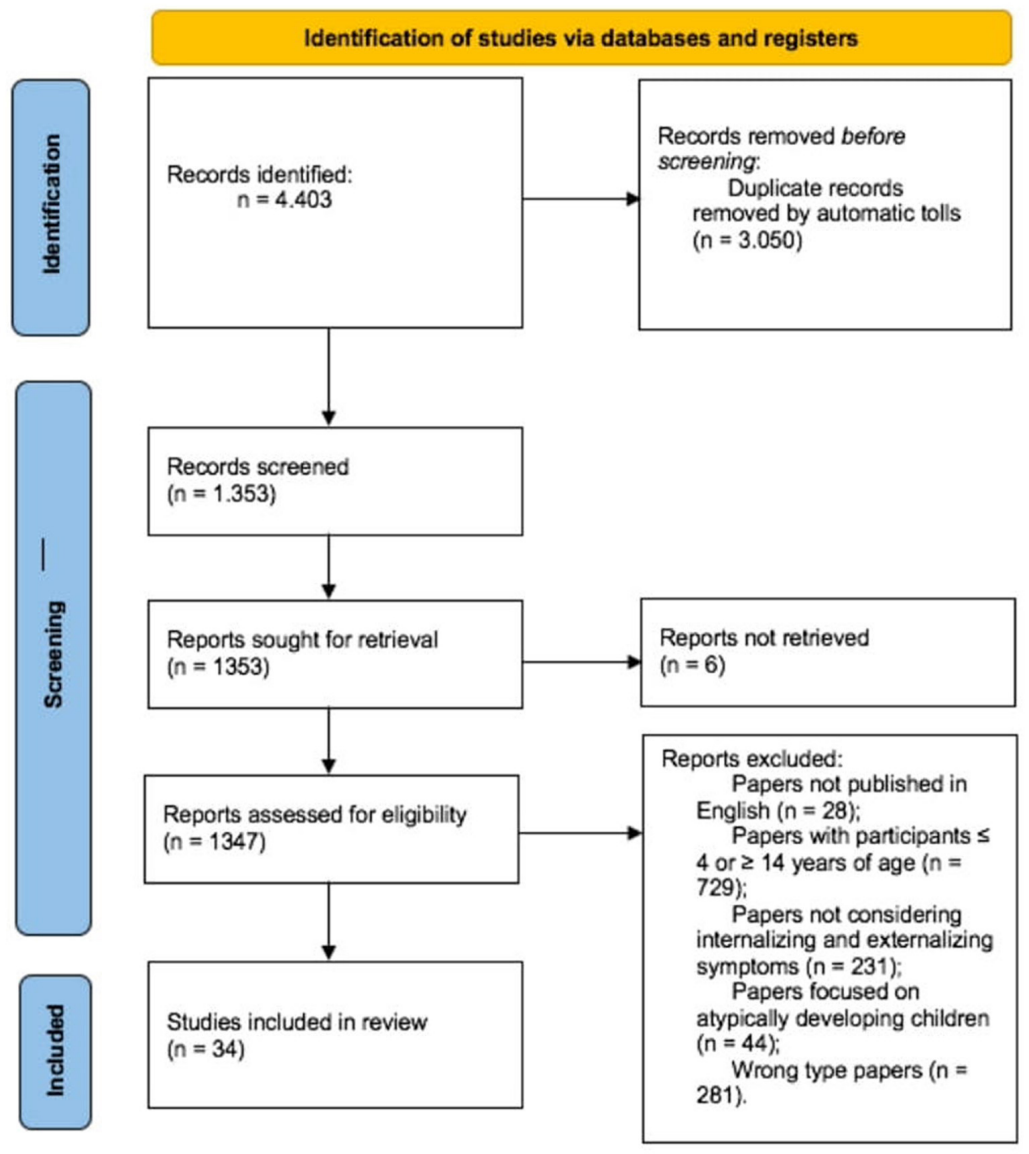
Flow chart of the selected studies.

Figure 1 maps the selection process. Following the Identification stage of PRISMA, we searched papers in which our keywords appeared in either the title, abstract, subject heading, or keywords list. Each keyword combination was tabulated in an Excel spreadsheet and 4,403 records were ordered alphabetically. All duplicates (*n* = 3,050) were removed.

A total of 1,353 papers were screened for the availability of the full text by two of the present authors (AL & CM); six papers were excluded because the full text could not be retrieved. A total of 1,347 papers were assessed and a total of 1,313 papers were excluded. Figure 1 details the number of papers that were excluded for each criterion. The inter-rater agreement was calculated using a set of 50 randomly selected papers; these were independently screened by AL and CM, and their disagreements were arbitrated by a third author (FL). The inter-rater agreement was good (Cohen's κ = 0.93). Thirty-four papers were included in the final review.

### 2.3. Quality appraisal

The quality of the papers was evaluated in accordance with the updated standardized Mixed Method Appraisal Tool protocol (MMAT; Hong et al., 2018). This protocol is used to evaluate five principal categories: qualitative studies (via four items), randomized control trials (via four items), non-randomized studies (via four items), quantitative descriptive studies (via four items), and mixed methods studies (via three items). For each category, a set of five questions are provided; a 3-point Likert scale (1 = *yes* and 0 = *no* and *can't tell*) is used to measure responses.

The MMAT comprises a spreadsheet into which the reviewer first inserts the study information (a reference ID number, the first author of the study, the publication year, and the full citation). The reviewer then selects from a drop-down menu the answer (*yes* vs. *no* vs. *can't tell*) to two preliminary screening questions (“Are there clear research questions?” and “Do the collected data allow to address the research questions?”). If *no* or *can't tell* are selected, the paper is excluded from the review. If *yes* is selected, the reviewer answers the five questions pertaining to the category of study.

In the present case, the inter-rater agreement was calculated using a set of 10 randomly selected papers. They were independently evaluated by two authors (AL & CM) and the disagreements were arbitrated by a third (FL). The inter-rater agreement was good (Cohen's κ = 0.95).

To calculate the overall score regarding the quality of α study, the MMAT developers suggest summing up the responses. They do not, however, recommend a cut-off point to categorize the overall score, arguing that reviewers make their own decision. We ranked the papers as low when they were rated as a 1 or 2, medium when they were rated as a 3, and high when they were rated as a 4 or 5.

### 2.4. Data synthesis

Because the results of the studies were heterogenous, a narrative approach was deemed appropriate. Section 3 herein comprises six parts. First, we provide an overview of the methodological characteristics of the studies (Section 3.1); the next four sections synthesize the findings and address the present study's research questions (Sections 3.2, 3.3, 3.4, and 3.5); and the sixth section summarizes the result of the quality appraisal (Section 3.6).

## 3. Results

Details of the main studies are tabulated in Table 1. For each study, we extracted the country and the time the data were collected; the overall methodology (i.e., quantitative vs. qualitative vs. mixed studies and cross-sectional vs. longitudinal); the strategies used to recruit the participants (probabilistic vs. non-probabilistic method and the specific strategy used); the method used to collect the data (online vs. face-to-face); and the individuals who completed the survey (parent vs. child). We also reported the sample's characteristics [i.e., the size of the total sample and the sub-samples (when present), gender distribution, mean age, standard deviation, and age range] and the outcome measures administered to evaluate children's internalizing and externalizing symptoms, the psychological determinants that were associated with or contributed to them, and any parent-related psychological determinants.

**Table 1:** Methodological characteristics of the included studies and quality appraisal.

**References**	**Country**	**Period of data collection**	**Study design^a^**	**Participants**		**Outcome measures^b^**	**Relevant findings**	**Study appraisal^c^**
				**Total sample** ***n*** **(% females)**	***M*** **(sd); age range**			
**Quantitative studies**								
Andrés et al. (2022)	Argentina	n.s.	Quantitative; cross-sectional; non-probabilistic sampling (i.e., snowball); online survey; parent/caregiver-report.	Total sample *n* = 1,205 (51.5% females). Sub-sample 6–8 yo *n* = 286 (% gender n.s.). Sub-sample 9–11 yo *n* = 297 (% gender n.s.).	Total sample *M*(*sd*) = n.s.; Age range = 3–18 yo. Sub-sample 6–8 yo *M*(*sd*) = n.s.; Age range = 6–8 yo. Sub-sample 9–11 yo *M*(*sd*) = n.s.; Age range = 9–11 yo.	(1) Child Behavior CheckList (Achenbach and Rescorla, 2014): child' internalizing and externalizing symptoms; Positive and Negative Affect Schedule for Children (Positive Affect Subscale; Laurent et al., 1999): child' positive affect. (3) State-trait Anxiety Inventory (Spielberger et al., 1999): parent' anxiety; Beck Depression Inventory-II (Beck et al., 1996): parent' depression; Positive and Negative Affect Schedule (López-Gómez et al., 2015): parent' affectivity; *Ad hoc* measure: parent' concerns and worry on COVID-19 infection.	RQ1 Children aged 6–8 years showed a high level of internalizing (anxiety-depression) and externalizing (impulsivity-inattention; aggression-irritability) symptoms. Children aged 9–11 years showed internalizing (anxiety-depression) and externalizing (aggression-irritability) symptoms. RQ2 Total sample Females > males: internalizing (anxiety and depression symptoms) symptoms; Males > females: externalizing (aggression and irritability) symptoms: Sub-sample 8–9 yo Males > females: dependence-withdrawal.	Medium
Dodd et al. (2022)	Ireland, UK	Irish sample April 3–April 26, 2020 UK sample April 4–April 15, 2020	Sub-sample Irish Quantitative; cross-sectional; non-probabilistic sampling (i.e., snowball); online survey; parent-report. Sub-sample UK Quantitative; cross-sectional; probabilistic sampling; online survey; parent-report.	Sub-sample Irish (Study 1) *n* = 427 (45% females). Sub-sample UK (Study 2) *n* = 1,919 (49% females).	Sub-sample Irish (Study 1) *M*(*sd*) = 8.02 (1.98) yo; Age range = 5–11 yo. Sub-sample UK (Study 2) *M*(*sd*) = 8.45 (1.99) yo; Age range = 5–11 yo.	Irish and UK samples (1) Strength and Difficulties Questionnaire (Goodman, 2003; Tobia and Marzocchi, 2018): child' internalizing and externalizing symptoms; Positive and Negative Affect Schedule for Children-P (Ebesutani et al., 2012): child' emotional functioning. (2) Children's Play Scale (Dodd et al., 2021): child' play. (3) Kessler-6 (Kessler et al., 2002): parent' distress.	RQ1 Irish and UK sample Child' internalizing symptoms were negatively associated with play activities; furthermore, low levels of child' internalizing symptoms were associated with low parent' distress levels. No significant associations between considered variables and child externalizing symptoms were found. RQ2 No significant gender differences in internalizing and externalizing symptoms were found. Females > males: positive affect. RQ3 The more child play activities involved the more positive effects.	Irish sample: High UK sample: High
Lionetti et al. (2022)	Italy	T1: January, 2020 T2: April, 2020	Quantitative; longitudinal; non-probabilistic sampling (convenience); online survey; parent-report.	*n* = 94 (55% females).	*M*(*sd*) = 9.08 (0.56) yo; Age range: 8–10 yo.	T1 (1) Pediatric Symptoms Checklist (Gardner et al., 1999): child' externalizing symptoms. (2) HSC scale (Pluess et al., 2018): child' environmental sensitivity. T2 (1) Pediatric Symptoms Checklist (Gardner et al., 1999): child' externalizing symptoms; (2) Closeness Scale of the Parent-Child Relationship Scale (Pianta, 1992): quality of parent-child relationship.	RQ1 Sensitive children showed more internalizing symptoms during the pandemic than before. RQ2 No gender differences in internalizing and externalizing symptoms were evaluated. RQ4 A close parent-child relationship moderated the impact of time on child' externalizing symptoms. Furthermore, the close parent-child relationship leads sensitive children to show decreased internalizing symptoms during the pandemic.	Low
Liu et al. (2022)	China	April 23–May 7, 2020.	Quantitative; cross-sectional; non-probabilistic sampling (convenience); online survey; self-report.	Total sample *n* = 4,852 (51.5% females). Sub-sample 10–12 yo *n* = 1,524 (49.5% females).	Total sample *M*(*sd*) = 13.80 (2.38) yo; Age range = 10–18 yo. Sub-sample 10–12 yo *M*(*sd*) = 10.96 (0.82) yo; Age range = 10–12 yo.	(1) Chinese version of Self-Rating Depression Scale (Zung, 1965): child' depressive symptoms; Chinese version of Self-Rating Anxiety Scale (Zung, 1971): child' anxiety symptoms. (2) Chinese Internet Addiction Scale-Revised (Chen et al., 2003): child' internet addiction; Chinese version of Athens Insomnia Scale (Soldatos et al., 2000; Chiang et al., 2009): child' insomnia; Chinese version of Utrecht Work Engagement Scale for Students (Schaufeli and Salanova, 2007; Fang et al., 2008): child' academic engagement.	RQ1 Depression and insomnia, as well as anxiety and insomnia mediated the relationship between problematic internet use and academic engagement. The indirect effects of Internet risk on academic engagement through depression and insomnia in middle and late adolescence were stronger than those in early adolescence; the direct effect in early adolescence was stronger than that in middle adolescence. RQ2 Females > males: internalizing (depression, anxiety) symptoms; Females > males: insomnia. RQ3 The older, female, and non-only children were significantly correlated with higher levels of internalizing (depression, anxiety and insomnia) symptoms.	High
Martiny et al. (2022)	Norway	June 8–July 3, 2020	Quantitative; cross-sectional; non-probabilistic sampling (snowball); online survey; parent and child -report.	*n* = 87 (51.7% females)	*M*(*sd*) = 9.66 (1.77) yo; Age range = 6-13 yo.	(1) How I feel Questionnaire, used with children as young as 8 years of age (Walden et al., 2003): child' internalizing symptoms. (2) *Ad hoc* measure: child' COVID-19 attitudes; KIDSCREEN-10 (Haraldstad et al., 2006; Ravens-Sieberer et al., 2006): child' wellbeing. (3) World Health Organization Index (Topp et al., 2015): parent' wellbeing; *Ad hoc* measure: parent' stress because of the reopening.	RQ1 Results show that high levels of child' wellbeing and positive emotions were associated with child' positive attitude toward the COVID-19. RQ2 No gender differences in internalizing and externalizing symptoms were evaluated. RQ4 Living with one parent was associated with low child' wellbeing; mother' wellbeing was associated with child' wellbeing and child' negative emotions.	Low
Morelli et al. (2022)	Italy	April, 2020	Quantitative; cross-sectional; non-probabilistic sampling (convenience); online survey; parent-report.	*n* = 277 (52% females).	*M*(*sd*) = 9.66 (2.29) yo; Age range = 6–13 yo.	(1) Emotion Regulation Checklist (Molina et al., 2014): child' emotions regulation. (2) *Ad hoc* measure: familiar risks related to the family situation during the lockdown, risks related to the COVID-19 pandemic, child' exposure to news related to COVID-19. (3) Modified version of the Television Mediation Scale (Valkenburg et al., 1999): parental mediation of children' exposure to news related to the COVID-19 pandemic.	RQ1 Results show an increase in anxiety and sadness in children. High level of child' emotion regulation and low level of lability/negativity were associated with parental active mediation style; low level of child' lability/negativity was associated with the parental restrictive style; child' lower emotion regulation was associated with parental social co-viewing style. RQ2 No significant gender differences in internalizing and externalizing symptoms were found. RQ3 Early adolescents show a lower level of emotion regulation than younger children.	Medium
Oliveira et al. (2022)	Portugal	June–July, 2020	Quantitative; cross-sectional; non-probabilistic sampling (snowball); online survey; parent-report.	*n* = 110 (50% females).	*M*(*sd*) = 9.09 (0.80) yo; Age range = 7–10 yo.	(1) Strength and Difficulties Questionnaire (Goodman, 2003): child' internalizing and externalizing symptoms. (2) KIDSCREEN-10 Index (Ravens-Sieberer et al., 2014): child' quality of life; Q25 Questionnaire (Oliveira et al., 2019): child' daily activities.	RQ1 Internalizing symptoms were positively correlated with domestic chores and negatively with play. Externalizing symptoms were positively correlated with gaming and negatively with creative leisure and play. Level of engagement in physical activities was positively correlated with psychological and social wellbeing and negatively with internalizing and externalizing symptoms. RQ2 No gender differences in internalizing and externalizing symptoms were evaluated. Males > females: physically active; Females > males: engaged in play and social activities. RQ3 There is evidence of high levels of sedentary behavior (time spent on the screen) and low levels of play and recreation, particularly among socioeconomically vulnerable children.	Low
Penner et al. (2022)	USA	February 2–April 4, 2021	Quantitative; cross-sectional; non-probabilistic sampling (quota); online survey; parent-reported.	Total sample *n* = 796 (42.1% females). Sub-sample 5–8 yo *n* = n.s. (% gender distribution n.s.). Sub-sample 9–12 yo *n* = n.s. (% gender distribution n.s.).	Total sample *M*(*sd*) = 10.35 (3.16) yo; Age range = 5–16 yo. Sub-sample 5–8 yo *M*(*sd*) = n.s.; Age range = 5–8 yo. Sub-sample 9–12 yo *M*(*sd*) = n.s.; Age range = 9–12 yo.	(1) Strength and Difficulties Questionnaire (Goodman, 2003): child' internalizing and externalizing symptoms. (2) Child Routines Inventory (Daily Living Routines subscale; Sytsma et al., 2001): child' daily routines; Part 1 (Exposures) of the COVID-19 Exposure and Family Impact Survey (Kazak et al., 2021): family COVID-19 exposure. (3) Short Forms of the Patient- Reported Outcomes Measurement Information System (PROMIS)-Depression and PROMIS-Anxiety (Pilkonis et al., 2011): parent' current depressive and anxiety symptoms; Short form of the Alabama Parenting Questionnaire (Elgar et al., 2007): parenting behaviors; Multidimensional Assessment of Parenting Scale (Hostility and Supportiveness subscales; Parent and Forehand, 2017): affective aspects of parenting; Parenting Sense of Competence Scale (Efficacy subscale; Johnston and Mash, 2010): parenting cognitions.	RQ1 For internalizing and externalizing symptoms, indirect associations occurred through increased parental hostility and inconsistent discipline and decreased parental routines and support. A negative correlation was found between child' internalizing symptoms and levels of positive reinforcement, daily routine, parental support and parental self-efficacy. A negative correlation was found between child' externalizing symptoms and levels of positive reinforcement, daily routine, parental support and parental self-efficacy. RQ2 No gender differences in internalizing and externalizing symptoms were found. RQ4 A positive correlation was found between high levels of inconsistent discipline, poor supervision and parental hostility.	Medium
Ravens-Sieberer et al. (2022)	Germany	May 26–June 10, 2020	Quantitative; cross-sectional; non-probabilistic sampling (convenience); online survey; parent–report (sub-sample 7–10 yo); self-report (sub-sample 11–13 yo).	Total sample *n* = 1,586 (50% females). Sub-sample 7–10 yo *n* = 546 (% gender distribution n.s.). Sub-sample 11–13 yo *n* = 351 (% gender distribution n.s.).	Total sample *M*(*sd*) = 12.25 (3.30) yo; Age range = 7–17 yo. Sub-sample 7–10 yo *M*(*sd*) = n.s.; Age range = 7–10 yo; Sub-sample 10–13 yo *M*(*sd*) = n.s.; Age range = 10–13 yo.	(1) Strength and Difficulties Questionnaire (Goodman, 2003): child' internalizing and externalizing symptoms; Selected items from the German version of the Center for Epidemiological Studies Depression Scale (Barkmann et al., 2008): child' depression symptoms; Screen for Child Anxiety Related Disorders (Birmaher et al., 1999): child' anxiety symptoms. (2) *Ad hoc* measure: child' burden of the pandemic; KIDSCREEN-10 Index (Ravens-Sieberer et al., 2014): child' quality of life; HBSC symptom check-list (Haugland et al., 2001): child' psychosomatic complaints.	RQ1 During the pandemic, children experienced high levels of anxiety, hyperactivity symptoms and peer problems. RQ2 Males > females: internalizing and externalizing symptoms. Females > males: externalizing symptoms (only for peer problems subscale). RQ3 During the pandemic, children experienced lower health-related quality of life than before the pandemic.	Medium
Sun et al. (2022)	USA	T1 Spring, 2019 T2 Spring, 2020	Quantitative; longitudinal; non-probabilistic sampling (convenience); T1: data were collected at school; T2: data were collected online.	*n* = 247 (47% females)	*M*(*sd*) = 8.13 (0.46 yo); Age range = 7–9 yo.	T1 (1) Teacher-Child Rating Scale (Perkins and Hightower, 2002): child' pre-pandemic social-emotional skills. T2 (1) Pediatric Emotional Distress Scale (three subscales; Saylor et al., 1999): child' internalizing and externalizing symptoms. (3) Center for the Epidemiological Studies of Depression Short Form (Björgvinsson et al., 2013): parent' depression symptoms; Generalized Anxiety Disorder 7- Item Scale (Spitzer et al., 2006; Löwe et al., 2008): parent' anxiety symptoms; UCLA Loneliness Scale version 3 (Russell, 1996): parent' loneliness; Brief Resilience Scale (Smith et al., 2008): parent' resilience.	RQ1 Results show that at the beginning of the pandemic, parents reported more children' externalizing symptoms than internalizing ones. Ability in relationships with peers before the pandemic predicted the child' internalizing and externalizing symptoms at pandemic onset. Child' externalizing symptoms were predicted by parental distress. RQ2 No gender differences in internalizing and externalizing symptoms were evaluated.	Low
Andrés-Romero et al. (2021)	Spain	Started in the third week of confinement until the sixth week (3 weeks)	Quantitative; cross-sectional; non-probabilistic sampling (snowball); online survey; parent-report.	Total sample *n* = 1,555 (53.18% females). Sub-sample 6–11 yo *n* = 353 (% gender distribution n.s.).	Total sample *M*(*sd*) = n.s.; Age range = 3–18 yo. Sub-sample 6–11 yo *M*(*sd*) = n.s.; Age range = 6–11 yo.	(1) Strength and Difficulties Questionnaire (Goodman, 2003): child' internalizing and externalizing symptoms. (2) *Ad hoc* measure: child' habits of everyday living. (3) Parental Stress Scale (Oronoz Artola et al., 2007): parent' stress; Resilience Scale (Wagnild, 2009): parent' resilience.	RQ1 High parental resilience levels were associated with low child' difficulties in terms of internalizing and externalizing symptoms. RQ2 No gender differences in internalizing and externalizing symptoms were evaluated. RQ3 Parents perceive a change in their child' habits and psychological difficulties.	Medium
Balayar and Langlais (2022)	USA	n.s.	Quantitative; cross-sectional [comparison before (retrospective) and during pandemic]; non-probabilistic sampling (snowball); online survey; parent-report.	*n* = 80 (50% females).	*M*(*sd*) = 8.7 (6.67) yo; Age range = 8–13 yo.	(1) *Ad hoc* measure: child' internalizing and externalizing symptoms. (2) *Ad hoc* measure: child' learning performance and psychosocial activities before and during the pandemic. (3) Depression, anxiety, stress scale (Henry and Crawford, 2005): parent' distress.	RQ1 Internalizing symptoms (withdrawn, anxious, depressed, and stressed) were significantly poorer during the pandemic than before. Regarding the externalizing symptoms, no significant differences before and during the pandemic were found. RQ2 No gender differences in internalizing and externalizing symptoms were evaluated. RQ3 Children' learning attainment during the pandemic was significantly predicted by externalizing symptoms.	Low
Bate et al. (2021)	USA	March 31–May 15, 2020	Quantitative study; cross-sectional; non-probabilistic sampling (snowball); online survey; parent-report.	*n* = 158 (43% females).	*M*(*sd*) = 8.73 (2.01) yo; Age range = 6–12 yo.	(1) Pediatric Symptoms Checklist (Jellinek et al., 1999): child' internalizing and externalizing symptoms; Child Revised Impact of Event Scale-13 (Perrin et al., 2005): child' trauma-related symptoms. (2) Child-parent relationship scale (Pianta, 1992): parent-child relationship quality. (3) *Ad hoc* measure: COVID-19 impact on parent; Patient Health Questionnaire (Spitzer et al., 1999): parent' emotional health; Impact of Events Scale -Revised (Weiss, 2007): parent' evaluation of own distress caused by traumatic events.	RQ1 Child' internalizing and externalizing symptoms were positively predicted by parent' emotional problems. RQ2 Females > males: internalizing symptoms; Males > females: externalizing symptoms. RQ4 The more conflictual parent-child relationship, the more the child' internalizing symptoms.	Low
Bianco et al. (2021)	Italy	April 1–May 4, 2020	Quantitative; cross-sectional; non-probabilistic sampling (snowball); online survey; parent-report.	*n* = 305 (49.5% females).	Females *M*(*sd*) = 10.58 (2.3) yo; Males *M*(*sd*) = 10.01 (2.4) yo; Age range = 6–13 yo.	(1) Child Behavior CheckList 6–18 years (Achenbach and Rescorla, 2014): child' internalizing and externalizing symptoms. (3) *Ad hoc* measure: COVID-19 exposure: parental exposure to COVID-19; Depression Anxiety Stress Scale−21 (Fonagy et al., 2016): parent' distress; Reflective Functioning Questionnaire (Fonagy et al., 2016): parent' reflective function.	RQ1 Child Internalizing symptoms (anxious/depressed) was associated with high maternal distress level and hypermentalization; child externalizing (attention problems, aggressive behavior) symptoms, were associated with high maternal distress level and hypermentalization. Child internalizing (anxious/depressed) symptoms and externalizing (attention problems, aggressive behavior) symptoms were associated with maternal exposure to COVID-19 infection. RQ2 Females > males: internalizing (anxiety and depression symptoms) symptoms.	High
Cellini et al. (2021)	Italy	April 1–April 9, 2020	Quantitative; cross-sectional; non-probabilistic sampling (snowball); online survey; parent-report.	*n* = 299 (46% females).	*M*(*sd*) = 7.96 (1.36) yo; Age range = 6–10 yo.	(1) Strength and Difficulties Questionnaire (Goodman, 2003): child' internalizing and externalizing symptoms. (2) Sleep Disturbance Scale for Children (Bruni et al., 1996): child' quality of sleep; Three items from Porcelli et al. (2018) and one item from Zakay (2014): child' time perception. (3) Pittsburgh Sleep Quality Index (Curcio et al., 2013): mother' quality of sleep; Subjective Time Questionnaire (Mioni et al., 2020): mother' perception of time; Strength and Difficulties Questionnaire−18+ (Goodman, 2003): parent' internalizing and externalizing symptoms (self-reported); Difficulties in Emotion Regulation (Giromini et al., 2012): parent' difficulties in emotional regulation.	RQ1 Results show an increase in three areas: (1) child' emotional symptoms; (2) child' conduct; (3) child' hyperactivity/inattention. RQ2 Males > females: hyperactivity-inattention, felt more bored; Females > males: poorer sleep. RQ3; RQ4 Low quality of sleep, children increasing boredom and the mother' emotional problems predicted children's emotional symptoms.	High
Khoury et al. (2021)	Canada	T0: 2016–2018; T1: May–November, 2020	Quantitative; longitudinal; non-probabilistic sampling (convenience); online survey; parent-report.	*n* = 68 (47.1% females).	*M*(*sd*) = 7.87 (0.75) yo; Age range = 7–9 yo.	(1) Brief Problem Monitor-Parent form for ages 6–18 years (Achenbach and Rescorla, 2014): child' externalizing symptoms. (2) Parent Behavior Inventory (Lovejoy et al., 1999): mother' behaviors over the past month; Center for Epidemiologic Studies Depression Scale (Andresen et al., 1994): mother' depressive symptoms over the past week; Generalized Anxiety Disorder-7 (Spitzer et al., 2006): mother' anxiety symptoms over the past 2 weeks; Perceived Stress Scale (Cohen, 1998): mother' experiences of stress over the past month.	RQ1 The results show an increase in internalizing and externalizing symptoms during the pandemic than before. Child' externalizing symptoms were associated with parental hostility; child' internalizing symptoms were associated with maternal anxiety. RQ2 No significant gender differences in internalizing and externalizing symptoms were found.	Low
Li and Zhou (2021)	China	February 28–March 5, 2020	Quantitative; cross-sectional; non-probabilistic sampling (snowball); online survey; parent-report.	Total sample *n* = 892 (47% females). Sub-sample 5–8 yo *n* = 647 (46% females). Sub-sample 9–13 yo *n* = 245 (51% females).	Total sample *M*(*sd*) = n.s.; Age range = 5–13 yo. Sub sample 5–8 yo *M*(*sd*) = 6.19 (0.99) yo; Age range = 5–8 yo; Sub-sample 9–13 yo *M*(*sd*) = 10.81 (1.40) yo; Age range = 9–13 yo.	(1) Spence Children's Anxiety Scale-Parent Version (Spence, 1999): child' internalizing symptoms; Early School Behavior Rating Scale (Caldwell and Pianta, 1991): child' externalizing symptoms. (2) *Ad hoc* measure: Family-Based Disaster Education Scale: disaster education provided by parents to their children during COVID-19. (3) Parental Worry Scale (Fisak et al., 2012): parent' worry in relation to their children during COVID-19.	RQ1 Child' internalizing and externalizing symptoms were associated with parental worry. RQ2 No gender differences in internalizing and externalizing symptoms were evaluated. RQ4 For the schoolchildren group only, fewer internalizing symptoms were associated with disaster-based education.	High
Liu et al. (2021)	China	February 25–March 8, 2020	Quantitative; cross-sectional; non-probabilistic sampling (snowball); online survey; parent-report.	Total sample *n* = 1,264 (44% females). Sub-sample Huangshi *n* = 790 (% gender distribution n.s). Sub-sample Wuhan *N* = 474 (% gender distribution n.s).	Total sample *M*(*sd*) = 9.81 (1.44) yo; Age range = 7–12 yo. Sub-sample Huangshi *M*(*sd*) = n.s.; Age range = 7–12 yo. Sub-sample Wuhan *M*(*sd*) = n.s.; Age range = 7–12 yo.	(1) Strength and Difficulties Questionnaire (Du et al., 2008): child' internalizing and externalizing symptoms. (3) Self-Rating Anxiety Scale (Zung, 1971): parent' anxiety symptoms.	RQ1 Children in Wuhan had more externalizing symptoms (problems with peers) and general difficulties than children in Huangshi. Children aged 10–12 yo had more externalizing symptoms in terms of problems with peers than children aged 7–9 yo. Children aged 7–9 yo had more externalizing symptoms in terms of problems in prosocial behaviors than children aged 10–12 yo. RQ2 Females > males: externalizing symptoms (peer problems). RQ4 Parental anxiety was associated with emotional symptoms in children.	Low
Mariani Wigley et al. (2021)	Italy	May 18–June 4, 2020	Quantitative; cross-sectional; non-probabilistic sampling (snowball); online survey; parent-report.	*n* = 158 (53% females).	*M*(*sd*) = 8.88 (1.41) yo; Age range = 6–11 yo.	(1) *Ad hoc* measure: child' internalizing and externalizing symptoms (stress-related behaviors, e.g., nervousness and irritability, difficulty falling asleep). (2) Child and Youth Resilience Measure-Revised (Personal Resilience subscale of the Person Most Knowledgeable version; Jefferies et al., 2019): child' individual resources. (3) *COPEWithME* questionnaire (developed in the study): parental teaching of resilient behaviors in children; Italian version of the Connor-Davidson Resilience Scale (Connor and Davidson, 2003): parent' resilience.	RQ1 Results show an increase in all child' stress-related behaviors (e.g., nervousness and irritability; difficulty falling asleep) investigated. RQ2 No gender differences in internalizing and externalizing symptoms were evaluated. RQ4 The greater was parental resilience, the better were the strategies used by parents to teach the child to manage stressful situations.	Low
Orgilés Amorós et al. (2021)	Italy; Spain; Portugal.	Seven weeks after the lockdown (15 days).	Quantitative; cross-sectional; non-probabilistic sampling (snowball); online survey; parent-report.	Total sample *n* = 515 (46% females). Sub-sample 6–12 yo *n* = 233 (gender distribution n.s.).	Total sample *M*(*sd*) = 8.98 (4.29) yo; Age range = 5–18 yo. Sub-sample 6–12 yo *M*(*sd*) = n.s.; Age range = 6–12 yo.	(1) Spence Children's Anxiety Scale-Parent Version (Spence, 1999): child' anxiety symptoms; Short Mood and Feelings Questionnaire-Parent Version (Angold et al., 1995): child' depressive symptoms. (2) *Ad hoc* measure: parent' stress due to the COVID-19 situation.	RQ1 The results show a high level of anxiety and depression in Spain. Italian children were more likely to present internalizing symptoms (depressive symptoms) than the Portuguese children. Internalizing symptoms (anxiety and depressive symptoms) were more likely in children whose parents reported higher levels of stress. RQ2 No gender differences in internalizing and externalizing symptoms were evaluated.	Low
Rajabi et al. (2021)	Iran	n.s.	Quantitative; cross-sectional; non-probabilistic sampling (snowball); online survey; parent-report.	*n* = 1,182 (44.5% females).	*M*(*sd*) = 7.18 (2.02) yo; Age range = 5–11 yo.	(1) Strengths and Difficulties Questionnaire–Parent version (Goodman, 2003): child' internalizing and externalizing symptoms; The International Positive and Negative Affect Schedule Short Form (Thompson, 2007): child' positive and negative affect. (2) Children's Play Scale (Dodd et al., 2021): child' time spent playing.	RQ1 Results show a significant negative correlation between mental health difficulties (internalizing and externalizing symptoms and positive and negative affect) and time spent playing. RQ2 Total sample Males > females: negative affect; Females > males: positive affect. Sub-sample 5–10 yo Males > females: Strength and Difficulties Questionnaire total score, emotional symptoms, hyperactivity/inattention subscales; Females > males: prosocial behavior subscale. Sub-sample 8–11 yo Males > females: emotional symptoms, hyperactivity/inattention, conduct problems, general problems subscales. Females > males: problems with peers and prosocial behavior subscales. RQ3 During COVID-19 children spent more time playing in the home setting and less time playing outdoors.	High
Scaini et al. (2021)	Italy	n. s.	Quantitative; cross-sectional; non-probabilistic sampling (snowball); online survey; parent-reported.	*n* = 158 (52% females).	*M*(*sd*) = 7.4 (1.8) yo; Age range = 5–10 yo.	(1) Strength and Difficulties Questionnaire (Goodman, 2003): child' internalizing and externalizing symptoms. (2) The Child and Youth Resilience Measure—Person Most Knowledgeable version (Ungar and Liebenberg, 2011): child' resilience; Junior Temperament and Character Inventory (Luby et al., 1999; Italian Version by Andriola et al., 2012): child' temperament and character.	RQ1 Child' externalizing symptoms were associated with low levels of persistence and reward dependence; internalizing symptoms were more likely among children with high harm avoidance and low persistence. RQ2 No significant gender differences in internalizing and externalizing symptoms were found. RQ3 High levels of resilience were associated with high levels of persistence and reward dependence.	Low
Vira and Skoog (2021)	Sweden	T1: October 2019–January, 2020; T2: November 2020–February, 2021.	Quantitative; longitudinal; non-probabilistic sampling (convenience); T1: data were collected at school; T2: data were collected via online survey; self-report.	*N* = 849 (51.83% females).	T1 *M*(*sd*) = 10 (0.03) yo; Age range = 9–11 yo. T2 *M*(*sd*) = 11 (0.05) yo; Age range = 10–12 yo.	(1) Strengths and Difficulties Questionnaire (subscale of emotional problems; Lundh et al., 2008): child' internalizing symptoms. (2) The Children's Hope Scale (Snyder et al., 1997): child' sense of hope; *Ad hoc* measure: Children's Self-Efficacy Scale: child' ability to be assertive and expressive; The Single-Item Self-Esteem Scale (SISE; Robins et al., 2001): child' self-esteem; Perceived Social Support (parents, close friends and teacher subscales; Malecki and Elliott, 1999): child' perceived social support; *Ad hoc* measure: child' school and class wellbeing.	RQ1 There were no significant differences in children' internalizing symptoms between T1 and T2. RQ2 No significant gender differences in internalizing and externalizing symptoms were found. RQ3 The results show a decrease in all factors assessed, in particular, children' perceived low support from teachers and class; low school wellbeing and self-esteem.	Medium
Wang et al. (2021a)	China	June 26–July 6, 2020	Quantitative; cross-sectional; probabilistic sampling (cluster method); online survey; parent-report.	*N* = 6,017 (45.4% females).	*M*(*sd*) = n.s.; Age range = 5–13 yo.	(1) Strength and Difficulties Questionnaire (Goodman, 2003): child' internalizing and externalizing symptoms. (2) *Ad hoc* measure: child' knowledge and precaution levels regarding COVID-19. (3) Depression Anxiety Stress Scale (Henry and Crawford, 2005): parent' distress; *Ad hoc* measure: parent' knowledge and precaution levels regarding COVID-19.	RQ1 Few child' emotional and behavioral symptoms were associated with increased knowledge and precautions regarding COVID-19 pandemic. Child' internalizing and externalizing symptoms were associated with parent' distress. RQ2 Males > females: Strength and Difficulties Questionnaire total score.	High
Wang et al. (2021c)	China	May 20–July 20, 2020	Quantitative; cross-sectional; probabilistic sampling (cluster method); online survey; parent-report.	Total sample *n* = 12,186 (47.8% females). Sub-sample Wuhan 6–11 yo *n* = n.s. (% gender distribution n.s.). Sub-sample outside Wuhan 6–11 yo *n* = n.s. (% gender distribution n.s.).	Total sample *M*(*sd*) = n.s.; Age range = 6–11 yo. Sub-sample Wuhan 6–11 yo *M*(*sd*) = 9.3 (1.43) yo; Age range = 6–11 yo. Sub-sample outside Wuhan 6–11 yo *M*(*sd*) = 9.1 (1.33); Age range = 6–11 yo.	(1) Child Behavior CheckList (Achenbach and Rescorla, 2014): child' internalizing and externalizing symptoms. (2) *Ad hoc* measure: psychosocial impact of pandemic on child.	RQ1 Children from Wuhan reported higher levels of schizoid and depression than children from outside Wuhan. RQ2 No significant gender differences in internalizing and externalizing symptoms were found.	High
Wang et al. (2021b)	China	June 26–July 6, 2020	Quantitative; cross-sectional; probabilistic sampling (cluster method); online survey; parent-report.	*n* = 6,017 (% gender distribution n.s.).	*M*(*sd*) = n.s. Age range = 5–13 yo.	(1) Strength and Difficulties Questionnaire (Stone et al., 2010): child' internalizing and externalizing symptoms. (2) *Ad hoc* measure: child' psychological stressors, daily activities, social interactions.	RQ1 The prevalence of externalizing symptoms (low prosocial behavior) was 17.85%. RQ2 Males > females: Strength and Difficulties Questionnaire total score. RQ3 Time used in homework and computer games was positively related to child' mental health problems; child' physical exercises were negatively related to frequency of communication with others.	Medium
Duan et al. (2020)	China	n.s.	Quantitative; cross-sectional; non-probabilistic sampling (convenience); online survey; self-report.	Total sample *n* = 3,613 (49.85% females). Sub-sample 7–12 yo *n* = 359 (% gender distribution n.s)	Total sample *M*(*sd*) = n.s.; Age range = 7–18 yo. Sub-sample 7–12 yo *M*(*sd*) = n.s.; Age range = 7–12 yo.	(1) Chinese Version of Spence Child Anxiety Scale (Zhao et al., 2012): child' anxiety symptoms; The Child Depression Inventory (Kovacs and Beck, 1977): child' depression symptoms. (2) *Ad hoc* measure: COVID-19 related questions (e.g., degree of concerns, implementation of control measures); Short Version of Smartphone Addiction Scale (Kwon et al., 2013) and Internet Addiction Scale from DSM-IV-TR (American Psychiatric Association, 2000): child' smartphone addiction; Coping Style Scale (Chen et al., 2000): child' coping strategies.	RQ1 The results show above-threshold results for depressive symptoms. RQ2 Females > males: internalizing (anxiety symptoms) symptoms. RQ3 The results show above-threshold results for internet addiction. The more time spent on the Internet, the higher the level of depressive symptoms.	Medium
Liang et al. (2020)	Italy	March 26–April 12, 2020	Quantitative; cross-sectional; non-probabilistic sampling (snowball); online survey; parent-report.	*n* = 1,074 (48% females)	*M*(*sd*) = 8.99 (1.97) yo; Age range = 6–12 yo.	(1) Impact Scale of the COVID-19 and home confinement on children and adolescents (Orgilés et al., 2020): child' internalizing and externalizing symptoms. (2) 11 items included the three dimensions proposed by Parker and Endler (Parker and Endler, 1992): child' coping strategies.	RQ1 The results show that children from Northern Italy were scared and they had greater fear of death than children from Central Italy. No significant differences regarding internalizing and externalizing symptoms between children from Northern and Central Italy were found. RQ2 No gender differences in internalizing and externalizing symptoms were evaluated. RQ3 Regarding coping strategies, children from Northern Italy used emotion-oriented coping strategies, while children from Central Italy used task-oriented coping strategies.	Medium
Morelli et al. (2020)	Italy	April, 2020	Quantitative; cross-sectional; non-probabilistic sampling (snowball); online survey; parent-report.	*n* = 233 (52% females).	*M*(*sd*) = 9.66 (2.29) yo; Age range = 6–13 yo.	(1) Emotion Regulation Checklist (Molina et al., 2014): child' emotions regulations. (2) *Ad hoc* measure: familiar risks related to the family situation during the COVID-19 pandemic. (3) Perceived Stress Scale (Cohen et al., 1983); Italian validation by Mondo et al. (2021): parent' distress; Regulatory Emotional Self-Efficacy Scale (Caprara et al., 2013): parental belief to be able to manage with their negative emotions; Parenting Self-Agency Measures (Dumka et al., 1996; Baiocco et al., 2017): parental belief to be able to manage with daily parental demands.	RQ1 Parental self-efficacy mediated the relationship between the influences of parent' psychological distress and parent' emotional regulatory self-efficacy on children' emotional regulation and lability/negativity. RQ2 No significant gender differences in internalizing and externalizing symptoms were found.	Medium
Petrocchi et al. (2020)	Italy	April 1–May 4, 2020	Quantitative; cross-sectional; non-probabilistic sampling (snowball); online survey; parent-report.	*n* = 144 (43% females).	*M*(*sd*) = 7.54 (1.6) yo; Age range = 5–10 yo.	(1) *Ad hoc* measure: child' internalizing symptoms (emotional responses); *Ad hoc* measure: child' adaptive behaviors. (2) *Ad hoc* measure: mother' exposure to COVID-19. (3) Depression Anxiety Stress Scale-−21 (Fonagy et al., 2016): mother' distress; Coping Scale (Hamby et al., 2015): mother' coping strategies.	RQ1 Child' internalizing symptoms (negative emotions) were associated with high maternal distress and low maternal coping strategies. RQ2 No gender differences in internalizing and externalizing symptoms were evaluated. RQ4 Mothers exposed to COVID-19 infection showed high distress levels and more coping strategies than mothers not exposed to virus infection.	Medium
**Qualitative studies**								
Aras Kemer (2022)	Turkey	n.s.	Qualitative; cross-sectional; non-probabilistic sampling (snowball); online survey; self-reported.	*n* = 9 (66% females).	*M*(*sd*) = n.s.; Age range = 7–10 yo.	(1) *Ad hoc* measure: child' anxiety evaluated via drawings and interviews.	RQ1 Drawings and interviews revealed internalizing symptoms (anxiety, negative emotions). RQ2 No gender differences in internalizing and externalizing symptoms were evaluated. RQ3 Results showed limited knowledge of the COVID-19 pandemic in children.	High
Cortés-García et al. (2021)	USA	May, 2020	Qualitative; cross-sectional; probabilistic sampling (random); online focus group; self-report.	Total sample *n* = 17 (52.9% females). Sub-sample 10–12 yo *n* = 9 (44.44% females).	Total sample *M*(*sd*) = n.s; Age range = 10–14 yo. Sub-sample 10–12 yo *M*(*sd*) = n.s; Age range = 10–12 yo.	(1) *Ad hoc* measure: semi-structured interview about child' emotional responses and coping strategies during pandemic.	RQ1 The results were mixed. On the one hand, children experienced positive feelings such as happiness (spending more time with parents, more free time and to play), on the other hand, children experienced negative feelings such as loneliness, sadness, boredom and fear (due to lack of socialization with friends and other family members). RQ2 No gender differences in internalizing and externalizing symptoms were evaluated. RQ3, RQ4 Other revealed themes were perception of racism, perception of economic impact and information related to COVID-19, quality of relationships in the family, use of coping strategies.	High
Idoiaga et al. (2020)	Spain	March 30–April 13, 2020	Qualitative; cross-sectional; non-probabilistic sampling (convenience); online open-ended questions; self-report.	Total sample *n* = 228 (52.21% females). Sub-sample 3–12 yo *n* = n.s. (% gender distribution n.s.).	Total sample *M*(*sd*) = 7.14 (2.57) yo; Age range = 3–12; Sub-sample 3–12 yo *M*(*sd*) = n.s. Age range = 6–12 yo.	(1)*Ad hoc* measure: open-ended questions about child' social and emotional representation of COVID-19.	RQ1 Results were mixed. On the one hand, they say they are bored, angry, overwhelmed, tired and even lonely because they have to stay at home without being able to go out. On the other hand, they also say they are happy and cheerful in the family. RQ2 No gender differences in internalizing and externalizing symptoms were evaluated. RQ3 Parents identified sibling relationship as particularly positive. Also, a disturbed sleep routine is reported.	Low
**Mixed study**								
Wenter et al. (2022)	Austria	T1: March/April, 2020 T2: December 2020/ January, 2021 T3: June/July, 2021 T4: December 2021/ January 2022.	Mixed study (convergent design); longitudinal; non-probabilistic sampling (convenience); online survey; parent-report.	Total sample *n* = 2.691 (48.8% females). Sub-sample 7–13 yo *n* = 1,740 (49.8% females).	Total sample *M*(*sd*) = n.s.; Age range = 3–13 yo. Sub-sample 7–13 yo *M*(*sd*) = 9.6 (1.9) yo; Age range = 7–13 yo.	(1) Child and Adolescent Trauma screen—caregiver report (Sachser et al., 2017): child' risk of post-traumatic stress disorder (Quantitative study); Child Behavior CheckList (Achenbach and Rescorla, 2014): child' internalizing and externalizing symptoms (Quantitative study); Kiddy-KINDL (Ravens-Sieberer and Bullinger, 2000): child' quality of life (Quantitative study). (2) *Ad hoc* measure: parent' evaluation of child exposure to COVID-19 infection (Quantitative study); *Ad hoc* measure: parent' evaluation of child' threat experience of COVID-19 (Quantitative study). (3) *Ad hoc* open-ended questions: parent description on the positive effects related to the COVID-19 pandemic (Qualitative study).	RQ1 Quantitative results: Data collected during the T4 wave showed a clinical classification of internalizing (emotional reactivity, anxious/depressed; somatic complaints; withdrawn/depressed) and externalizing (aggressive behaviors) symptoms. Qualitative results: Thematic analysis showed that the themes were: importance of intra- and extra-familiar relationships; new competence and experiences; values and virtues; use of time; and family strengths. RQ2 Males > females: externalizing (aggressive behaviors) symptoms. RQ3 Threat experience increased internalizing and externalizing symptoms, post-traumatic symptoms, and low quality of life.	Medium

^a^Study design (Qualitative vs. quantitative vs. mixed study) (cross-sectional vs. longitudinal); sampling strategy; data collection strategy (online vs. face-to-face); respondent (parent- vs. self-report).

^b^Measures: (1) measure(s) administered to evaluate the child' internalizing/externalizing symptoms; (2) measure(s) administered to evaluate other(s) child' psychological factor(s); (3) measure(s) administered to evaluate parent(s) psychological factor(s).

^c^Study appraisal: ^*^ and ^**^ = low; ^***^ = medium; ^****^ and ^*****^ = high.

n.s, not specified.

^*^ and ^*^^*^ mean that the quality of the paper is low; ^*^^*^^*^ means that the quality of the paper is medium; ^*^^*^^*^^*^ and ^*^^*^^*^^*^^*^ mean that the quality of the paper is high.

We then summarized the relevant findings of each study and organized them according to the research questions. We reported the main results (RQ1), the psychological determinants (RQ2), gender differences (RQ3), and the parental role (RQ4). In the last column of Table 1, we report the quality appraisal for each study (low vs. medium vs. high) based on the MMAT protocol.

### 3.1. Methodological characteristics

A total of 34 studies were included in this systematic mixed studies review. They derived from 15 countries across three Continents (Europe, America, and Asia). The majority (*n* = 18) were European, nine were Asian, and seven were American (*n* = 6 North America; *n* = 1 South America). Table 1 is structured according to the category of the research design: quantitative (*n* = 30), qualitative (*n* = 3), and mixed studies (*n* = 1).

Except for five quantitative studies (Duan et al., 2020; Rajabi et al., 2021; Scaini et al., 2021; Andrés et al., 2022; Balayar and Langlais, 2022) and one qualitative study (Aras Kemer, 2022), the papers provided information on when the data were collected. Most of them were quantitative and collected data during the first 7 months of 2020 (Liang et al., 2020; Morelli et al., 2020, 2022; Petrocchi et al., 2020; Andrés-Romero et al., 2021; Bate et al., 2021; Bianco et al., 2021; Cellini et al., 2021; Li and Zhou, 2021; Liu et al., 2021, 2022; Mariani Wigley et al., 2021; Wang et al., 2021a,b,c; Dodd et al., 2022; Lionetti et al., 2022; Martiny et al., 2022; Oliveira et al., 2022; Ravens-Sieberer et al., 2022), as did the qualitative studies (Idoiaga et al., 2020; Cortés-García et al., 2021). One quantitative study (Penner et al., 2022) collected data during the first months of 2021 (February–April).

Most of the papers applied a cross-sectional quantitative study design (*n* = 27); three collected longitudinal quantitative data. All three qualitative studies were cross-sectional. The mixed study applied a longitudinal design for the quantitative section and used cross-sectional data for the qualitative section. Of the four longitudinal studies (Khoury et al., 2021; Vira and Skoog, 2021; Sun et al., 2022; Wenter et al., 2022), three (Khoury et al., 2021; Vira and Skoog, 2021; Sun et al., 2022) compared data collected before and during the pandemic. Lionetti (Lionetti et al., 2022) collected data in January 2020 and April 2020. Sun et al. (2022) compared data collected during Spring 2019 and Spring 2020. Khoury et al. (2021) compared data collected during 2016–2018 with those collected during May–November 2020. The mixed study included data collected during four pandemic waves between 2020 and 2022 (Table 1).

Eighty-five percent of the studies (*n* = 26 quantitative studies; *n* = 2 qualitative studies; *n* = 1 mixed study) recruited participants using non-probabilistic sampling strategies. The remaining studies (*n* = 4 quantitative studies; *n* = 1 qualitative study) used probabilistic strategies (Table 1).

As expected, because of the COVID-19 restrictions, all the studies collected data remotely, inviting participants to complete an e-survey disseminated through the main social platforms and/or mailing lists. Only two longitudinal studies (Vira and Skoog, 2021; Sun et al., 2022) collected data face-to-face (before the pandemic) and online (during the pandemic).

Most of the study questionnaires (*n* = 27) were completed by a parent or caregiver; two (Martiny et al., 2022; Ravens-Sieberer et al., 2022) were completed by both parents and children; and five (Duan et al., 2020; Idoiaga et al., 2020; Cortés-García et al., 2021; Vira and Skoog, 2021; Aras Kemer, 2022) were completed by the children. A total of 40,976 participants were enrolled on the studies. For the 30 quantitative studies, the total sample ranged between 80 and 12,186 participants; for the three qualitative studies, it ranged between 9 and 228. The mixed study involved 2,691 participants.

It is worth noting that the majority of the studies enrolled children from a wider age range (e.g., 5–18). Because the present study is focused on middle childhood (i.e., children aged 5–13), we extrapolated information regarding the size of the sub-group(s). In the quantitative studies, the sub-groups varied from 233 to 1,919 participants; two of the qualitative studies divided the total sample into sub-groups, and only one reported the number (*n* = 9). The sub-group in the mixed study comprised 1,740 participants.

We extracted information on the gender distribution percentage for each study. The majority of the quantitative studies (*n* = 21) reported the gender distribution percentage for both the total and sub-groups (where applicable); eight studies (Andrés-Romero et al., 2021; Liu et al., 2021; Orgilés Amorós et al., 2021; Wang et al., 2021b; Andrés et al., 2022; Penner et al., 2022; Ravens-Sieberer et al., 2022) reported the gender percentage for the total sample only. One study (Wang et al., 2021c) did not report the gender distribution. All the quantitative studies were balanced, as was the mixed study. Two qualitative studies (which were similarly balanced) provided detailed information on gender, while one study (Idoiaga et al., 2020) did not offer any.

We also calculated the participants' mean age, standard deviations, and age range(s), though this was not possible for six quantitative studies (Duan et al., 2020; Andrés-Romero et al., 2021; Wang et al., 2021a,b,c; Andrés et al., 2022) because the necessary information was not available. Of the quantitative studies that split the total samples into sub-groups, seven did not report the above details (Duan et al., 2020; Andrés-Romero et al., 2021; Liu et al., 2021; Orgilés Amorós et al., 2021; Andrés et al., 2022; Penner et al., 2022; Ravens-Sieberer et al., 2022). Three of the qualitative studies (Idoiaga et al., 2020; Cortés-García et al., 2021; Aras Kemer, 2022) did not report the participants' ages. Finally, the mixed design study authors did not provide the mean ages and the standard deviations of the total sample, though they did for the sub-samples.

Table 1 displays information on the measures administered by the authors of the studies. We filed the outcome measures according to the psychological construct(s): the measure(s) assessing children's internalizing and externalizing symptoms; the tool(s) evaluating the children's psychological determinant(s); and the measure(s) assessing the parent-related psychological determinants(s). For each measure, we point out the full name, reference, and the psychological construct that was evaluated.

The majority of the quantitative studies applied validated measures; most applied the Strength and Difficulties Questionnaire (Goodman, 2003). Three papers (Petrocchi et al., 2020; Mariani Wigley et al., 2021; Balayar and Langlais, 2022) applied non-validated measures. To evaluate the children's psychological determinant(s), 11 (Liang et al., 2020; Bate et al., 2021; Cellini et al., 2021; Mariani Wigley et al., 2021; Rajabi et al., 2021; Scaini et al., 2021; Dodd et al., 2022; Lionetti et al., 2022; Liu et al., 2022; Oliveira et al., 2022; Penner et al., 2022) applied validated measures only, nine (Morelli et al., 2020, 2022; Petrocchi et al., 2020; Andrés-Romero et al., 2021; Li and Zhou, 2021; Wang et al., 2021a,b,c; Balayar and Langlais, 2022) applied non-validated measures only, and four (Duan et al., 2020; Vira and Skoog, 2021; Martiny et al., 2022; Ravens-Sieberer et al., 2022) applied validated and non-validated measures. To evaluate parental psychological determinants, 25 applied validated measures, five (Bate et al., 2021; Bianco et al., 2021; Wang et al., 2021a; Andrés et al., 2022; Martiny et al., 2022) used both validated and non-validated measures, and one (Orgilés Amorós et al., 2021) applied a non-validated measure. All of the qualitative studies assessed children's internalizing and externalizing symptoms using non-validated measures; the children's and parents' psychological determinants were not evaluated. Finally, the mixed study applied validated measures to evaluate children's internalizing and externalizing symptoms and non-validated measures to assess children's and parent's psychological determinants.

### 3.2. The impact of the COVID-19 pandemic on children's internalizing and externalizing symptoms

The present section addresses RQ1. As Table 1 shows, one study (Wang et al., 2021c) estimated that 17.85% of participants were above the threshold for externalizing symptoms only. The longitudinal studies revealed that the levels of both internalizing (Khoury et al., 2021; Lionetti et al., 2022) and externalizing (Khoury et al., 2021) symptoms were higher during the pandemic than they were previously. One longitudinal study (Sun et al., 2022) reported that the levels of externalizing symptoms were higher than internalizing ones during the pandemic. Only one study (Vira and Skoog, 2021) found no difference before and during the pandemic. The quantitative cross-sectional studies reported high levels of internalizing (Duan et al., 2020; Liang et al., 2020; Cellini et al., 2021; Orgilés Amorós et al., 2021; Andrés et al., 2022; Morelli et al., 2022; Ravens-Sieberer et al., 2022) and externalizing (Cellini et al., 2021; Mariani Wigley et al., 2021; Andrés et al., 2022; Ravens-Sieberer et al., 2022) symptoms compared with the threshold. By contrast, one study (Balayar and Langlais, 2022) reported low levels of both types of symptoms during the pandemic compared with the period before. The qualitative studies generated mixed results. Two suggested that children experienced low (Idoiaga et al., 2020; Cortés-García et al., 2021) levels of internalizing symptoms, while one (Aras Kemer, 2022) suggested the opposite. Finally, the quantitative results of the longitudinal mixed study demonstrated clinical scores (i.e., over the threshold) for internalizing and externalizing symptoms. The qualitative data of the mixed study did not focus on children's internalizing and externalizing symptoms.

### 3.3. The psychological determinants associated with or contributing to children's internalizing and externalizing symptoms

The present section addresses RQ2. We analyzed the associations between children's internalizing/externalizing symptoms and their relevant psychological determinant(s). Several quantitative studies demonstrated that high levels of children's internalizing (Petrocchi et al., 2020; Rajabi et al., 2021; Oliveira et al., 2022) and externalizing symptoms (Rajabi et al., 2021; Oliveira et al., 2022) were associated with low engagement during play activities. Furthermore, more widespread use of the internet during the pandemic led to high levels of internalizing symptoms (Duan et al., 2020; Liu et al., 2022). The qualitative studies did not examine this issue. The mixed studies revealed that the constant recommendations and restrictions imposed during the lockdowns increased children's internalizing and externalizing symptoms.

### 3.4. Gender differences between children's internalizing and externalizing symptoms

The present section addresses RQ3. The 30 quantitative studies reported mixed findings. Some studies (Duan et al., 2020; Bate et al., 2021; Bianco et al., 2021; Andrés et al., 2022; Liu et al., 2022) found that female children reached higher levels of internalizing symptoms than their male peers, with only two studies (Wang et al., 2021a,c) showing the opposite. Three studies (Bate et al., 2021; Rajabi et al., 2021; Andrés et al., 2022) reported that male children showed more externalizing symptoms than their female peers. One study (Liu et al., 2021) indicated that female children reached higher levels of externalizing symptoms than males. Six studies (Morelli et al., 2020, 2022; Scaini et al., 2021; Wang et al., 2021b; Dodd et al., 2022; Penner et al., 2022) observed no difference, and 10 studies (Liang et al., 2020; Petrocchi et al., 2020; Andrés-Romero et al., 2021; Li and Zhou, 2021; Mariani Wigley et al., 2021; Orgilés Amorós et al., 2021; Balayar and Langlais, 2022; Martiny et al., 2022; Oliveira et al., 2022; Sun et al., 2022) did not investigate gender.

These included the qualitative studies. Finally, the quantitative findings of the mixed study revealed that male children showed more externalizing symptoms than females. The qualitative findings of the study did not examine gender differences.

### 3.5. Parental psychological determinants influencing children's internalizing and externalizing symptoms

The present section addresses RQ4. The 30 quantitative studies examined the associations between parental psychological determinants and children's internalizing/externalizing symptoms. They concluded that several parental psychological determinants, for example, distress (Petrocchi et al., 2020; Bianco et al., 2021; Orgilés Amorós et al., 2021; Wang et al., 2021a; Andrés et al., 2022), hostility (Cellini et al., 2021), and emotional difficulties (Bate et al., 2021; Cellini et al., 2021; Liu et al., 2021) affected their children's internalizing symptoms. In addition, parental self-efficacy, inconsistent discipline (Bronfenbrenner, 1979), conflictual parent–child relationships (Bate et al., 2021), hypermentalizing (Walden et al., 2003), worries (Li and Zhou, 2021), and poor ability to cope with stressful situations (Petrocchi et al., 2020) were negatively associated with children's internalizing symptoms. Similarly, high hostility and inconsistent discipline (Penner et al., 2022), low self-efficacy (Penner et al., 2022), emotional problems (Bate et al., 2021; Cellini et al., 2021), high distress and problems with hypermentalizing (Bianco et al., 2021), and low parental resilience were associated with children's externalizing symptoms.

The results of the quantitative longitudinal studies emphasized that positive and non-conflictual parent–child relationships moderated the degree of change in externalizing symptoms (Lionetti et al., 2022). The results also stressed that children's externalizing symptoms were predicted by parental distress (Sun et al., 2022) and that they were associated with parental hostility (Khoury et al., 2021). One study (Khoury et al., 2021) showed that internalizing symptoms were associated with maternal anxiety. Finally, the qualitative and mixed studies did not consider the influence of parental psychological determinants on children's internalizing and externalizing symptoms.

### 3.6. Quality appraisal

The quality appraisal of the studies using the MMAT protocol concluded that they were either low (*n* = 12), medium (*n* = 12), or high (*n* = 10). As was pointed out in Section 2.3, the quality of each study was regarded as low when it was rated as a one or two, medium when it was rated as a three, and high when it was rated as a four or five. The details of each study appraisal are tabulated in Table 2 (It should be noted before proceeding that the MMAT protocol takes a rather conservative approach to quality appraisal).

**Table 2:** Details of the quality appraisal of the reviewed studies.

	**Screening questions**		**Items**					
**References**	**S1. Are there clear research questions?**	**S2. Do the collected data allow to address the research questions?**	**4.1. Is the sampling strategy relevant to address the research question?**	**4.2. Is the sample representative of the target population?**	**4.3. Are the measurements appropriate?**	**4.4. Is the risk of non-response bias low?**	**4.5. Is the statistical analysis appropriate to answer the research question?**	**Quality appraisal**
**Quantitative studies**								
Andrés et al. (2022)	Yes	Yes	Can't tell	Yes	Yes	Can't tell	Yes	Medium
Dodd et al. (2022) (Irish sample)	Yes	Yes	No	Yes	Yes	Yes	Yes	High
Dodd et al. (2022) (UK sample)	Yes	Yes	Yes	Yes	Yes	Yes	Yes	High
Lionetti et al. (2022)	Yes	Yes	No	No	Yes	Can't tell	No	Low
Liu et al. (2022)	Yes	Yes	No	Yes	Yes	Yes	Yes	High
Martiny et al. (2022)	Yes	Yes	No	Yes	Yes	Can't tell	Yes	Medium
Morelli et al. (2022)	Yes	Yes	No	Yes	Yes	No	Yes	Medium
Oliveira et al. (2022)	Yes	Yes	No	No	Yes	No	Yes	Low
Penner et al. (2022)	Yes	Yes	No	Yes	Can't tell	Yes	Yes	Medium
Ravens-Sieberer et al. (2022)	Yes	Yes	No	Yes	Yes	Can't tell	Yes	Medium
Sun et al. (2022)	Yes	Yes	No	Yes	No	No	Yes	Low
Andrés-Romero et al. (2021)	Yes	Yes	No	Yes	Yes	Can't tell	Yes	Medium
Balayar and Langlais (2022)	Yes	Yes	No	Yes	Can't tell	Can't tell	Yes	Low
Bate et al. (2021)	Yes	Yes	No	No	Yes	Can't tell	Yes	Low
Bianco et al. (2021)	Yes	Yes	No	Yes	Yes	Yes	Yes	High
Cellini et al. (2021)	Yes	Yes	No	Yes	Yes	Yes	Yes	High
Khoury et al. (2021)	Yes	Yes	No	No	Yes	No	Yes	Low
Li and Zhou (2021)	Yes	Yes	No	Yes	Yes	Yes	Yes	High
Liu et al. (2021)	Yes	Yes	No	No	Yes	No	Yes	Low
Mariani Wigley et al. (2021)	Yes	Yes	No	No	Yes	Can't tell	Yes	Low
Orgilés Amorós et al. (2021)	Yes	Yes	No	No	Yes	Can't tell	No	Low
Rajabi et al. (2021)	Yes	Yes	No	Yes	Yes	Can't tell	Yes	Medium
Scaini et al. (2021)	Yes	Yes	No	No	Yes	Can't tell	Yes	Low
Vira and Skoog (2021)	Yes	Yes	No	Yes	No	Yes	Yes	Medium
Wang et al. (2021a)	Yes	Yes	Yes	Yes	Yes	Yes	Yes	High
Wang et al. (2021c)	Yes	Yes	No	Yes	Yes	Yes	Yes	High
Wang et al. (2021b)	Yes	Yes	Yes	Yes	No	Can't tell	Yes	Low
Duan et al. (2020)	Yes	Yes	No	Yes	Yes	Can't tell	Yes	Medium
Liang et al. (2020)	Yes	Yes	No	Yes	Yes	Can't tell	Yes	Medium
Morelli et al. (2020)	Yes	Yes	No	Yes	Yes	Yes	Yes	High
Petrocchi et al. (2022)	Yes	Yes	No	Yes	No	Yes	Yes	Medium
			**1.1. Is the qualitative approach appropriate to answer the research question?**	**1.2. Are the qualitative data collection methods adequate to address the research question?**	**1.3. Are the findings adequately derived from the data?**	**1.4. Is the interpretation of results sufficiently substantiated by data?**	**1.5. Is there coherence between qualitative data sources, collection, analysis and interpretation?**	**Quality appraisal**
**Qualitative studies**								
Aras Kemer (2022)	Yes	Yes	Yes	No	Yes	Yes	Yes	High
Cortés-García et al. (2021)	Yes	Yes	Yes	No	Yes	Yes	Yes	High
Idoiaga et al. (2020)	Yes	Yes	Yes	No	No	Yes	No	Low
**Mixed studies**								
Wenter et al. (2022)	Yes	Yes						
		**Qualitative study**	**1.1. Is the qualitative approach appropriate to answer the research question?**	**1.2. Are the qualitative data collection methods adequate to address the research question?**	**1.3. Are the findings adequately derived from the data?**	**1.4. Is the interpretation of results sufficiently substantiated by data?**	**1.5. Is there coherence between qualitative data sources, collection, analysis and interpretation?**	
			Yes	Yes	Yes	Yes	Yes	
		**Quantitative study**	**4.1. Is the sampling strategy relevant to address the research question?**	**4.2. Is the sample representative of the target population?**	**4.3. Are the measurements appropriate?**	**4.4. Is the risk of non-response bias low?**	**4.5. Is the statistical analysis appropriate to answer the research question?**	
			No	Yes	Yes	Can't tell	Yes	
		**Mixed study**	**5.1. Is there an adequate rationale for using a mixed methods design to address the research question?**	**5.2. Are the different components of the study effectively integrated to answer the research question?**	**5.3. Are the outputs of the integration of qualitative and quantitative components adequately interpreted?**	**5.4. Are divergences and inconsistencies between quantitative and qualitative results adequately addressed?**	**5.5. Do the different components of the study adhere to the quality criteria of each tradition of the methods involved?**	**Quality appraisal**
			Yes	Yes	Yes	Yes	Yes	Medium

First, the quantitative studies. Item 1 focuses on sampling strategy. As was expected, the preferred choice for the MMAT protocol is probabilistic sampling. For the quantitative studies, 28 used the main non-probabilistic sampling strategies; two (Wang et al., 2021a,c) did not. Item 2 examines whether the sample enrolled for a study is representative of the target population. The majority of the studies (*n* = 22) met the representativeness criteria proposed by the MMAT protocol (e.g., a clear description of any attempt to achieve the enrolled sample of participants represents the target population). Item 3 evaluates whether the measurements administered in a study are adequate to answer the research question(s). The majority of the studies (*n* = 24) applied appropriate, validated, and gold-standard measures. Item 4 assesses the risk of non-response bias. Just over one third of the studies (*n* = 11) met the criterion. In other words, they had a low non-response rate and/or they used statistical compensation for non-responses (e.g., the imputation method). Finally, Item 5 indicates whether the statistical plan is appropriate for answering the research question(s). All the studies, with the exception of two (Orgilés Amorós et al., 2021; Lionetti et al., 2022), clearly stated the statistical plan and adequately computed their analysis of the design and research question(s). The overall quality appraisal of the quantitative studies was low and medium in 11 cases and high in eight cases.

Secondly, the qualitative studies. Item 1 evaluates the suitability of a qualitative approach for answering research question(s). All the studies met this criterion. Item 2 appraises the adequacy of the data collection method. None of the studies used appropriate methods (e.g., validated interviews). For Item 3, which evaluates whether data collection methods are suitable, the appraisal revealed that two of the studies applied methods based on the theoretical framework; one (Idoiaga et al., 2020) did not. Item 4 is used to show whether the interpretation of the findings is supported by the data; all the qualitative studies met this criterion. Finally, Item 5 weighs the relationship between data collection, analysis, and interpretation. Two studies were coherent in this regard; one (Idoiaga et al., 2020) was not. The overall quality appraisal of the qualitative studies was low for one study and high for two.

Finally, because the mixed methods study involved a combination of quantitative and qualitative methods, the reviewer evaluated the quality of both independently, then evaluated the items of the mixed study section; the quality of the quantitative study was high and the quality of the qualitative study was medium (Table 2). The mixed study section met all the methodological quality criteria. It provided a sound rationale for using a mixed study method (Item 1). The quantitative and qualitative results were effectively integrated (Item 2), and a meta-inference (i.e., the overall interpretation derived from integrating the quantitative and qualitative results) was made. Finally, the quantitative and qualitative components did not diverge (Item 4), and the study was found to be trustworthy (Item 5).

According to the user guide provided by the MMAT developers, the quality of a mixed methods study depends on the quality of its quantitative and qualitative components. Therefore, its overall quality cannot exceed the quality of its weakest component. So, in the present instance, the overall quality appraisal was medium.

We were cognisant of two significant issues in our quality appraisal. First, six studies (Duan et al., 2020; Rajabi et al., 2021; Scaini et al., 2021; Andrés et al., 2022; Aras Kemer, 2022; Balayar and Langlais, 2022) did not state when they collected the data, so the research context was not clear. This was a serious flaw because the results could not be interpreted relative to the time frame when the data were collected. The second matter concerned the administration of non-validated measures. We considered this to be important in light of the seriousness of the subject of our research. Such a shortcoming is likely to lead scholars to approach certain findings with caution.

## 4. Discussion

The present study aimed to complement the literature by synthesizing and appraising evidence of the impact of the COVID-19 pandemic on children's internalizing and externalizing symptoms. In an attempt to address our research questions, we searched five databases, extracted information from 34, and summarized our findings using a narrative approach.

The studies were conducted in several countries, so it became evident that children's mental health is now a matter of global concern. As was expected, the majority of the studies collected data online because of the restrictions imposed by governments worldwide. Most of the studies reported parents' perceptions of their children's functioning using validated measures.

Albeit one study (Wang et al., 2021c) assessed the prevalence of externalizing symptoms only, the results of the 34 studies overall suggested that parents/caregivers were conscious that their children exhibited high levels of internalizing and externalizing symptoms in their children (RQ1). The longitudinal studies, which compared the levels of these symptoms before and during the pandemic, confirmed the negative impact of the COVID-19 pandemic on children's mental health (Khoury et al., 2021; Lionetti et al., 2022; Sun et al., 2022; Wenter et al., 2022). Moreover, an effective prohibition on play activities and an increase in internet use were the main psychological determinants influencing children's internalizing and externalizing symptoms (RQ2).

To better understand why the pandemic had detrimental effects on children's functioning, two studies applied Bronfenbrenner's ecological system theory (Bronfenbrenner, 1977, 1979). During the pandemic, children's microsystems, that is, their immediate environment (e.g., family and school), were damaged (Chachar et al., 2021) by school closures, isolation, and the subsequent cessation of social and peer relationships. Daily routines were disrupted, and this made children more vulnerable. Compromised microsystems created a negative association between children's internalizing/externalizing symptoms and their functioning and adaptive behaviors. Indeed, the results of the studies reviewed herein indicated that high levels of internalizing and externalizing symptoms were part of the reason children withdrew from play activities and began to spend more time online. Social isolation, home confinement, and a prohibition on outdoor play were associated with high levels of depressive symptoms, trait anxiety, aggressive behavior, irritability, and inattention. A lack of shared play during in-presence peer interactions may have led children to feel bored, whereupon they began to be habituated to their toys and engage less in their usual play activities at home. This, in turn, may have led them to play games online in search of enjoyable experiences and to maintain social contact with their peers, albeit virtually. However, because such activities are a poor substitute for face-to-face social interaction, they may have exacerbated children's internalizing and externalizing symptoms.

This vicious cycle has prompted us to reflect on the impact of the pandemic on children's functioning and adaptive behaviors in the context of the family. In particular, parents' fear of the contagion and their concerns thereof may have been conveyed to their children, with deleterious consequences for their wellbeing (i.e., high levels of internalizing and externalizing symptoms) and, in turn, their adaptive behaviors (i.e., play activities). Similarly, the high levels of children's internalizing and externalizing symptoms associated with the disruption of their daily routines and a lack of face-to-face social interactions may have affected their parents' wellbeing, in terms of high levels of distress and maladjustment. This strengthens the argument that the pandemic markedly affected reverse parent–child relationships and that intervention programs involving all family members might play a pivotal role in their recalibration.

The present study confirms the existence of gender differences in internalizing and externalizing symptoms (RQ3) that had been identified in the pre-pandemic era (Yang et al., 2008; Bender et al., 2012). Female children were significantly more vulnerable to internalizing symptoms (e.g., depression and anxiety) compared with their male counterparts. Meanwhile, the latter exhibited externalizing symptoms (e.g., aggressive behaviors, hyperactivity, and inattention) more than their female counterparts. While not all the studies agreed, some (Chen, 2010; Batra et al., 2020; Campbell et al., 2021) reported gender differences between children's internalizing and externalizing symptoms. The implication here is that mental health intervention programs should pay special attention to children's wellbeing and prevent them from developing these symptoms. Some of the studies observed no gender difference (*n* = 6) or discovered that female children showed more externalizing symptoms than their male counterparts (*n* = 3). This may be explained by the powerful emotional effects of the pandemic. Restrictive measures, disrupted routines, and so on may have disrupted the self-regulation and the self-control of children and pre-adolescents, regardless of gender. Future longitudinal studies might explore whether these findings derived from the extraordinary situation imposed by the pandemic, or whether other contextual factor(s) may have contributed to the way females and males responded to unforeseen environmental stimuli. Finally, because some studies were methodologically flawed by not taking gender into account, their results should be treated circumspectly.

All the studies revealed that children's functioning was associated with a range of parental psychological determinants (RQ4). The results indicated that children's internalizing symptoms reflected high levels of parental distress, while their externalizing symptoms were primarily a consequence of parental hostility and inconsistently applied discipline. Again, the ecological system theory could be a useful lens through which to examine this topic. For parents, pandemic-related stressors, such as have difficulties paying bills, juggling work and family obligations, managing children's home-schooling, taking care of older parents, and so on may have resulted in higher levels of distress and inefficient parenting. If individual protective psychological factors are low (e.g., parental resilience and/or efficient parenting), it is difficult to counter the negative psychological impact of such stressors. Indeed, they may have led to a deterioration in the parents' mental health and the parent–child relationship. Because the effects were reciprocal, intervention programs must be aimed at both parents and their children.

## 5. Conclusion

The present study has summarized crucial information on the impact of the COVID-19 pandemic on mental health during the middle childhood stage. It is hoped that it will serve as a wake-up call to governments and policy-makers when designing and providing targeted intervention programs to support children/pre-adolescents and their parents in future situations. Close attention should be paid to internalizing and externalizing symptoms that, when not measured or left untreated, could lead to significant adverse outcomes in children's subsequent developmental stages (Kim-Cohen et al., 2003). In brief, our results support the need for intervention programs that address children's and preadolescents' mental health and wellbeing in the present and future.

## 6. Strengths and limitations

The present study has several strengths. First, it provides information that might be applied in real-world contexts. The findings demonstrate that, between 2020 and 2022, high levels of internalizing and externalizing symptoms were experienced by children aged 5–13 years. This needs to be taken into account when intervention programs are being developed and/or updated so that the negative short- and long-term effects of the pandemic on children's mental health and wellbeing are mitigated. The findings also demonstrate that parental psychological determinants may have exacerbated children's internalizing and externalizing symptoms. For example, several of the studies showed that a close parent–child relationship can play a positive role in alleviating psychological and social distress (Bate et al., 2021; Lionetti et al., 2022). Therefore, coaching programs targeting parents' strengths and/or protective factors should be developed to help every family member cope with extraordinary environmental conditions.

Another strength of the present study lies in its methodology. First, to ensure methodological rigor, we applied four standardized protocols (PEO, PICOS, PRISMA, and MMAT). In addition, extracting detailed information from quantitative, qualitative, and mixed studies allowed us to significantly extend our knowledge of the internalizing and externalizing symptoms experienced by children aged 5–13 years between 2020 and 2022. Our results will help future researchers overcome the limits and gaps in the literature.

The present study has some limitations. First, although we searched five major databases, others may have yielded other relevant articles. Secondly, we did not search the gray literature. Thirdly, the majority of the studies used e-survey completed by parents; hence, the results should be read in light of parental perceptions of the children's functioning, because these may be prone to error and bias. These limitations should be addressed in future research.

## 7. Recommendations

We would like to make some clinical and research recommendations because, according to our findings, children's internalizing and externalizing symptoms and their psychological determinants increased during the pandemic. From a clinical perspective, mental health interventions should be designed for what is a vulnerable population. A recent meta-analysis (Jugovac et al., 2022) revealed that attachment- and emotion-focused interventions enable parents to recognize, understand, and respond to their children's emotional needs, so these could be useful in reducing internalizing and externalizing symptoms. They should also take children's gender differences into account, thus contributing to a novel line of research. Moreover, our results highlight the need to design bespoke coaching programs for parents that reduce distress and hostility and promote effective discipline and positive parent–child relationships. In short, government and state agencies responsible for mental health policy should prioritize children's and parents' mental health, directing their efforts toward mitigating the short- and long-term psychological effects of the COVID-19 pandemic.

The present study has several implications for future research. First, our findings revealed that the majority of the studies accessed their data cross-sectionally; it is therefore not yet known how children's internalizing and externalizing symptoms might affect them in the future. Hence, researchers should conduct longitudinal studies that collect data over the next months and beyond, so the effects of restrictive measures such as lockdowns and school closures on the functioning of children, adolescents, and their parents can be investigated in the short-, medium-, and long-term.

Secondly, future studies might validate the non-validated measures that were used in some of the studies so they can be used for other cohorts. Thirdly, two flaws in the MMAT protocol impacted the appraisal of the studies. Albeit probabilistic methods were impracticable during the pandemic, future researchers might endeavor to increase the generalizability of their findings. The second flaw relates to the application of compensatory strategies for non-responses. Authors would be advised to state in their papers the percentage of missing data and/or the method applied to limit the risk of bias (e.g., imputation or the removal of data). This would improve the quality of their studies and the interpretations thereof.

Finally, authors must report all necessary information. As has been noted, several of the studies did not state when they collected their data or the gender distribution of the participants. This is a significant omission because the studies included in the present review aimed to investigate children's functioning during a period that was punctuated by constant challenges and openings and closures. Scholars and clinicians must possess this information so they can more accurately assess the authors' findings. Given the existence of gender differences in internalizing and externalizing symptoms (Chen, 2010; Bender et al., 2012; Campbell et al., 2021), it is crucial that all authors clarify the composition of participants. Scholars must, therefore, apply strict methodological rigor to their studies. In sum, it is hoped that the above recommendations will be of benefit to future researchers, and subsequently, children and their parents.

## Data availability statement

The raw data supporting the conclusions of this article will be made available by the authors, without undue reservation.

## Author contributions

Conceptualization of the methodology: AL and FL. Searched for the scientific literature, screened the records, and appraisal each paper according to the standardized protocol: AL and CM. Writing the draft: AL. Tabulated information of the papers: CM. Arbitrated any methodological disagreements: FL. Conceptualization of the topic: All authors. All authors critically revised the manuscript and agreed with its submission in the current form.
